# Catquest-9SF questionnaire: Validation in a Greek-speaking population using Rasch analysis

**DOI:** 10.1371/journal.pone.0278683

**Published:** 2022-12-07

**Authors:** Panagiotis Nanos, Vasiliki Kouteliari, Eirini-Kanella Panagiotopoulou, Nikolaos Papadopoulos, Panagiota Ntonti, Georgios Labiris

**Affiliations:** 1 Department of Ophthalmology, General Hospital of Kalamata, Kalamata, Greece; 2 Department of Ophthalmology, University Hospital of Alexandroupolis, Dragana, Alexandroupolis, Greece; The Ohio State University, UNITED STATES

## Abstract

**Purpose:**

The Catquest-9SF questionnaire is a tool measuring visual disability and vision-related limitation in patients’ daily activities. The primary objective of this study was the validation of Catquest-9SF in a Greek-speaking cataract population.

**Methods:**

The questionnaire was translated into Greek and translated back into English. A pre-final Greek version was formed and tested by 10 Greek-English bilingual participants and by the translation team, and the final version was produced. Patients scheduled for cataract surgery completed the questionnaire preoperatively and postoperatively. Rasch analysis was performed for the assessment of the Catquest-9SF psychometric properties, including response category ordering, item fit statistics, principal components analysis, precision, differential item functioning and targeting for preoperative and postoperative data collectively.

**Results:**

A total of 100 (55 men, 45 women, mean age = 71.94±6.63) cataract patients completed the Greek version of Catquest-9SF questionnaire preoperatively and postoperatively. Rasch analysis showed a significant improvement in the median person Rasch score from −1.49 preoperatively to −4.71 logits postoperatively, while the effect size was 1.3. Unidimensionality was confirmed since infit and outfit mean square values varied between 0.66 and 1.37. Rasch analysis showed good precision and separation ability (Person Separation Index of 3.28, and Person Reliability of 0.92). Four response categories were found for all items. The item-person means difference was -1.83 logits. The difference between preoperative and postoperative Catquest-9SF logit score was positively correlated with preoperative Catquest-9SF logit score (coeff. = 0.798, p<0.0001) and negatively correlated with postoperative spherical equivalent (coeff. = -0.825, p = 0.011).

**Conclusion:**

The Greek version of Catquest-9SF proved to be reliable, valid, unidimensional and responsive to changes after cataract surgery presenting good psychometric properties for cataract patients. Some postoperative mistargeting was found indicating that the tasks were easily performed by respondents after cataract surgery.

**Trial registration:**

NCT05323526 -retrospectively registered.

## Introduction

Cataract is one of the most prevalent causes of vision loss, being responsible for approximately 33% of visual incapacity worldwide and 51% of blindness [1, 2]. Cataract surgery is a commonly performed operation with high efficacy and low complication rates [3–5]. For the best possible evaluation of cataract extraction surgery outcomes, the success of the operation should be measured both with objective, such as preoperative and postoperative visual acuity and residual refractive error, and subjective indices, such as patients’ satisfaction and their ability to perform activities of daily living using special questionnaires [6, 7]. It is worth noting that high expectations of some patients after an uneventful cataract surgery may lead to a moderate patient satisfaction despite the high postoperative visual acuity and the minimum refractive error, resulting in a moderate self-perceived visual function and vision-related quality of life [8, 9].

A variety of validated questionnaires developed for the quantitative assessment of cataract surgery outcomes and postoperative visual function are available [10]; among them, the Visual Functioning 14 (VF-14) [10], the Activities of Daily Vision Scale (ADVS) [11, 12], the Visual Disability Assessment (VDA) [13, 14], the Quality of life and Vision Function Questionnaire (QOL-VFQ) [15], and Cataract TyPE Specification (TyPE Spec) [16]. Another common validated questionnaire evaluating self-assessed visual function is the Catquest questionnaire [17]. This questionnaire is validated with Rasch analysis, which is the gold standard of validation methods, and is used to evaluate the benefit after cataract surgery. The Catquest questionnaire has been first developed and used in Sweden since 1995 [18], and, in 2009, a revised 9-item version (short-form), called the Catquest-9SF questionnaire, was validated [19]. Since then, it has been translated, culturally adapted, and evaluated, in chronological order, in Australia (2009) [20], Germany and Austria (2013) [21], Italy (2016) [22], Spain (2016) [23], the Netherlands (2017) [24], Denmark (2018) [25], England (2018) [26], China and Malaysia (2018) [27, 28], Canada (2020) [29], and France (2021) [30].

Although the Catquest questionnaire is available in a great variety of languages, it has been not translated and validated in Greek, yet. Within this context, the primary objective of this study was to translate and validate the Greek version of the Catquest-9SF questionnaire in a cataract population before and following a cataract extraction surgery, as well as to subsequently assess its psychometric properties via Rasch analysis.

## Methods

### Setting

This was a prospective questionnaire validation study. Study protocol adhered to the tenets of the Helsinki Declaration and written informed consent was obtained by all participants. The institutional review board of Democritus University of Thrace and of General Hospital of Kalamata (GHK) approved the protocol (S1–S4 Files). The study was conducted at the Department of Ophthalmology of University Hospital of Alexandroupolis (UHA), Alexandroupolis, Greece, and of GHK, Kalamata, Greece between February 2020 and December 2021. The official registration number of the study is NCT05323526.

### The Catquest-9SF questionnaire

The Catquest-9SF questionnaire is a Rasch-scaled tool measuring visual disability and limitation in patients’ activities of daily living (ADLs) because of their vision. It may be used in cataract patients before and after their cataract surgery. It consists of nine questions (items); 2 global assessment items (A and B) evaluating the patients’ general perception of difficulties and their satisfaction with vision, and 7 difficulty items related to perceived difficulty in performing specific ADLs (C1-C7). Each item has four response categories ranging from 1 (no difficulty) to 4 (very great difficulty). For the global question about the patients’ satisfaction with their vision, the response options range from 1 (very satisfied) to 4 (very dissatisfied). Each item has also a “Cannot decide” option which is treated as a missing answer in the Rasch analysis. The raw score is calculated summing the answers of the 9 items and ranges from 9 to 36 [19]. The Catquest-9SF is publicly available for all health care organizations through the International Consortium for Health Outcomes Measurement (ICHOM) standard sets for cataract, and a license is not needed for use.

### Translation process

The Catquest-9SF questionnaire was translated into the Greek language by a translation team consisting of five members: one translation coordinator, two professional native English-speaking translators, one professional native Greek-speaking translator and one native Greek ophthalmologist fluent in English. First, the Catquest-9SF questionnaire was translated from English [Australian version Gothwal VK et al. Clin Exp Ophthalmol. (2009)] to Greek by the professional native Greek-speaking translator and the native Greek ophthalmologist in an independent way (forward translation). Then, the two translators and translation coordination met to compare the two versions, resolve the discrepancies and produce a reconciled version. Subsequently, the Greek reconciled version of the Catquest-9SF was translated back to English by the two professional native English-speaking translators working independently (back translation). Finally, all members of the translation team met to review and compare the back translation with the English version of the Catquest-9SF. The arose differences were discussed and a pre-final Greek version of the Catquest-9SF questionnaire was formed. The pre-final version was tested in series of 10 Greek-English bilingual participants in the target population, who made comments on the translated version. Later, the results of all the discussions were reviewed and edited by the translation team and the final Greek version of the questionnaire was produced (S5 File).

### Participants

Patients were recruited from the Cataract Service of the Department of Ophthalmology in the UHA and the GHK, in a consecutive-if-eligible basis. Eligibility criteria included age over 18 years, diagnosis of unilateral or bilateral senile cataract with stage 2 nuclear opalescence according to the Lens Opacities Classification System III (LOCS-3) grading scale, and no severe cognitive impairment during their preoperative examination. To avoid statistical bias, data of only one eye of each patient was collected, even if they were scheduled for bilateral cataract surgery. Patients who were scheduled for cataract surgery in both eyes did not have surgery in the second eye within the study period. Patients with ocular and systemic comorbidities were included, since this is a typical characteristic of a cataract population. Exclusion criteria included difficulty with the Greek language or comprehension, neurological, psychiatric or mental diseases, and generally inability to understand the Catquest questions.

### Surgical technique

All patients underwent cataract extraction surgery by phacoemulsification with implantation of monofocal intraocular lenses. All operations were performed by the same surgeons (G.L. & P.N.) in a consistent way. Pupils were dilated with Tropicamide 0.5% (Tropixal, Demo, Greece), Cyclopentolate 1% (Cyclogyl, Alcon Laboratories Inc., Fort Worth, TX, USA) and Phenylephrine Hydrochloride 5% (Phenylephrine, Cooper, Greece). Periorbital skin and the lids were cleaned and the conjuctival cul-de-sac was irrigated with povidone iodine (Betadine). Patients received topical anesthesia with proparacaine hydrochloride 0.5% drops (3 drops prior to surgery).

The foldable hydrophilic acrylic monofocal intraocular lens enVista (Bauch & Lomb, Rochester, NY, USA) was inserted in the capsular bag, targeting −0.25 D. The same postoperative regimen was prescribed to all patients, which included fixed combination of chloramphenicol 0.1% and dexamethasone 0.5% (Dexachlor, Cooper, Greece) six times daily, and Sodium Hyaluronate 0.1% (Hylocomod, Pharmex, Greece) gradually tapered in a month.

### Data collection

The questionnaire was self-administered to Greek-speaking and writing participants. Patients responded to the Greek version of Catquest-9SF in the presence of an independent researcher who had no direct involvement in the provision of care. All questionnaires were completed and returned on the same day of the preoperative assessment prior to the clinical examination. The pre- and postoperative assessment was performed by the same ophthalmologist with no direct involvement in the study. Demographic and clinical data regarding the participants was retrieved from their medical records. Proxy responses (i.e. from family members) were excluded. Test-retest reliability for all Catquest-9SF items was assessed in all participants in two different visits with an average 15-day time window to prevent memory effect.

The following clinical parameters were evaluated: preoperative and postoperative best spectacle-corrected distance visual acuity (BSCDVA) of the eye scheduled for cataract surgery, binocular BSCDVA, as well as refraction, spherical equivalent (SE) and intraocular pressure (IOP) of the eye scheduled for cataract surgery.

### Rasch analysis

The characteristics of the Catquest-9SF data were assessed by Rasch analysis. Rasch analysis is a psychometric model based on modern test theory, which is extensively used for the assessment and improvement of existing questionnaires [12, 31–36], as well as for the construction of new ones [37–39]. In specific, Rasch analysis compares the level of difficulty that is required for respondents to perform a task as listed in the items (items’ difficulty) with the respondents’ level of ability to perform that task (respondents’ ability), and both are evaluated along the same linear scale. The ordinal raw scores of the data are transformed into linear, interval Rasch scores [37, 40], namely into a unit known as “logit”, which is the natural logarithm of the odds ratio. Then, rasch scores are summed to a total score for each respondent. This total score can be interpreted as a measure of functional ability [41]. A more negative logit value indicates a better score, namely, a person with a greater ability (lower disability), and an item with greater difficulty.

Rasch analysis assessed the Greek version of Catquest-9SF questionnaire for response category ordering, item fit statistics, principal components analysis, precision, differential item functioning and targeting. First, response category ordering was evaluated graphically with category probability curves by observing whether the four response categories—omitting ‘Cannot decide’ as missing—(three category thresholds) were utilized in ascending order.

Second, overall fit of the data to the model and unidimensionality were evaluated using item fit statistics [infit and outfit mean square (MNSQ)]. In specific, when data fits well, the items should contribute to a single construct (visual disability) indicating the unidimensionality of the questionnaire. Both infit and outfit mean squares have an expected value of 1.0, with an ideal range of 0.7 to 1.3, and an acceptable range of 0.5 to 1.5 [28, 40]. A value < 0.7 indicates too little variance and easily predicted questions, while > 1.3 indicates too much variance and less predicted questions than the model expects [24]. In addition to item statistics, unidimensionality was further assessed using principal components analysis (PCA) of the residuals. Unidimensionality indicates that the questionnaire measures the underlying trait (visual difficulty). Two criteria are used; the PCA assessment is performed by comparing the amount of variance explained empirically and by the model, and the amount of variance explained by the first contrast (additional dimension) [28, 42]. For unidimensionality, the unexplained variance in the first contrast of the residuals should be < 2.0 eigenvalue units, and ≥ 60% of variance should ideally be explained by the measure, while a percentage of ≥ 50% is acceptable [42, 43].

Third, measurement precision of the Greek Catquest-9SF was assessed in terms of Person Separation Index (PSI) and Person Reliability (PR). PSI implies the questionnaire’s ability to differentiate along its scale. The greater the value of person separation, the greater the questionnaire precision (acceptable person separation > 3.0). PR (ranging from 0 to 1) should be > 0.9 to be considered adequate, indicating a high ability to distinguish between the strata of person ability [44].

Fourth, the differences between different subgroups of patients, known as Differential Item Functioning (DIF), were assessed, since the questionnaire item calibration should be comparable across different patient groups. DIF occurs if patients from different subgroups with the same underlying true ability have an unequal probability of giving a response to an item [41]. DIF was assessed by evaluating age (patients ≥ 72 vs < 72 years old, 72 years was the mean age of our sample), gender (males vs females), existence of ocular comorbidities, planning for cataract surgery of the first or the second eye, binocular BSCDVA (≥ 0.15 vs < 0.15 logMAR, 0.15 logMAR was the median BSCDVA of the sample), and cataract surgery of the eye planned to be operated (preoperative vs postoperative data). DIF (namely DIF contrast defined as the difference of DIF size between two subgroups in logit) of > 1.0 logits is significant, DIF with values between 0.5 and 1.0 is minimal, and DIF < 0.5 logits is considered absent [29, 41, 45].

Finally, targeting of the questions to the patient sample, which indicates how well the items’ difficulties match the person ability [41], was assessed to establish if the questions are appropriate for patients before and following cataract surgery. This can be assessed graphically by observing the spread along the person-item map and by the difference between the item and the person mean values. A well-targeted instrument requires the ability of the patients and the difficulty of the questions to be centered on the same mean (targeting of 0). In general, a mean difference in magnitude of ≥ 1 logit indicates significant mistargeting, while a difference of < 1.0 logit indicates good targeting, with targeting of 0 being ideal [41, 45–47]. A positive value indicates that the items are too difficult relative to person’s ability, while a negative value indicates that the items are too easy. A person–item map also visualizes the item hierarchy of difficulty, ranging from a minimum to maximum difficulty of performance.

### Statistical analysis

Rasch analysis was performed on preoperative and postoperative data stacked as a single dataset. Only patients who responded to both general items (A and B) and at least five of the remaining seven Catquest-9SF items (C1–C7) were included in the data analysis. Catquest-9SF scores are derived via Rasch analysis and more negative (or less positive) scores suggest better visual disability. Test-retest reliability was assessed by calculating intraclass correlation coefficients (ICCs) (two-way mixed model with measures of absolute agreement—single measurement) for person and item measures (in logits) estimating stacked preoperative and postoperative data.

Data distribution of the questionnaire items was tested with Shapiro-Wilk test. Data was found to be non-normally distributed. Therefore, the count, the median and the interquartile range [1st quartile, 3rd quartile] were reported.

In addition, multiple stepwise regression analysis was performed. The dependent variable used was the algebraic difference in Catquest-9SF score (logit_preop_—logit_postop_) and the independent variables investigated were age, gender, preoperative and postoperative binocular BSCDVA and SE, as well as preoperative Catquest-9SF logit score. Moreover, Spearman correlation was applied between monocular BSCDVA of the eye scheduled to be operated/monocular BSCDVA of the fellow eye/binocular BSCDVA and person measures (logit score) both for preoperative and postoperative data.

Descriptive statistics were performed with the Medcalc version 20.1.4 (Medcalc Software, Mariakerke, Belgium), and Rasch analysis was performed using version 4.8.2 of Winsteps software (Chicago, IL, USA) [48, 49].

Ceiling and floor effects were identified; they were defined a priori as more than 15% of respondents achieving the best or worst possible logit score, respectively [50, 51]. Specifically, since a more negative logit value indicates a better score and a less negative logit value indicates a worse score, ceiling and floor effects are present when more that 15% of respondents achieve the most negative and most positive possible logit score, respectively. Sensitivity to change was assessed using a two-sided paired samples Wilcoxon test comparing pre- and postoperative Catquest-9SF raw and logit scores. P-values lower than 0.05 were considered statistically significant. The effect size was calculated as improvement (preoperative–postoperative) divided by the preoperative standard deviation in logit scores. Effect sizes of 0.80 or above were considered large [19, 52–56].

## Results

### Participant characteristics

A total of 100 patients (55 men, 45 women, 71.94 ± 6.63 years) were recruited and completed the translated Catquest-9SF questionnaire preoperatively and during the postoperative examination at 1 to 3 months after the cataract surgery (mean number of days after surgery: 60 ± 29.16 days) (Table 1), responding to items A and B and at least 5 of the remaining 7 Catquest-9SF items. Postoperative BSCDVA of the operated eye was significantly better than the preoperative values (p < 0.0001). A percentage of 49% of patients had pre-existing ocular comorbidity and 82% had systemic comorbidity. Among the ocular comorbidities of our cohort were glaucoma, pseudoexfoliation, corneal disease, macular or retinal disease, proliferative or non-proliferative diabetic retinopathy. One-eye cataract surgery had already been performed in 41 patients, while the remaining patients had not undergone any cataract surgery. Most of the patients in our study had finished their primary school (66.0%).

**Table 1:** Demographic characteristics. Pre- and postoperative clinical data.

Parameters	
**N [patients (eyes)]**	100
	55 male / 45 female
	47 right / 53 left
**Age (years) [mean ± SD]**	71.94 ± 6.63
**Eye surgery (first eye/second eye)** (N)	59 / 41
**Ocular comorbidity**^a^ **(Yes/No)** (N)	49 / 51
**Systemic comorbidity**^b^ **(Yes/No)** (N)	82 / 18
**Educational level** (N)	
Primary education	66
Lower secondary education	10
Upper secondary education	20
Higher education	4
**BSCDVA of the eye scheduled for cataract surgery (logMAR)**	
Median [interquartile range]	
preoperative	0.40 [0.30, 0.50]
postoperative	0.07 [0.00, 0.15]
p-value	< 0.0001*
**Binocular BSCDVA (logMAR**)	
Median [interquartile range]	
preoperative	0.15 [-0.004, 0.25]
postoperative	-0.004 [-0.05, 0.05]
p-value	< 0.0001*
**Spherical equivalent of the eye scheduled for cataract surgery (D)**	
Median [interquartile range]	
preoperative	-1.25 [-3.00, -1.38]
postoperative	-0.38 [-0.75, 0.15]
p-value	0.02*
**Intraocular pressure of the eye scheduled for cataract surgery (mmHg)**	
Median [interquartile range]	
preoperative	18.00 [11.00, 32.00]
postoperative	16.00 [10.00, 32.00]
p-value	<0.0001*

^a^Includes glaucoma, corneal disease, retinal disease, non-proliferative or proliferative diabetic retinopathy, pterygium, others

^b^Includes hypertension, diabetes mellitus, heart disease, hyperlipidaemia, others

*p-value < 0.05

D: Diopters, BSCDVA: Best Spectacle-Corrected Distance Visual Acuity, N: Number of participants, SD: Standard Deviation

Missing answers (or “Cannot decide” option) were always ≤ 5%, and no ceiling or floor effect was present preoperatively, however, there was a marked ceiling effect in the postoperative assessment with 16 out of 100 respondents (> 15%) to achieve the lowest possible logit score [50]. Taking into account both preoperative and postoperative data, no ceiling or floor effect was present (ceiling effect: 4.5%, floor effect: 1%).

Rasch analysis of the 100 pre- and postoperative questionnaires showed a significant improvement in the median person Rasch score (p < 0.0001), from −1.49 [-2.45, 2.17] logits to −4.71 [-5.73, -2.91] logits, using the Wilcoxon signed-rank test. The improvement was on average 4.06 ± 3.73 logits. The effect size was 1.3.

### Unidimensionality

Unidimensionality is a significant assumption of Rasch analysis. As aforementioned, item fit statistics (MNSQ) and PCA both indicate unidimensionality. Both the “infit” and the “outfit” MNSQ values for each item were acceptable (0.5–1.5). The infit mean square varied between 0.66 and 1.36, while the outfit mean square ranged from 0.66 to 1.37 (Table 2), suggesting the unidimensionality of the questionnaire. Further principal components analysis (PCA) testing revealed that the observed raw variance explained by the measures was 74.3% (> 50%), and the unexplained variance in the first, second, third, fourth and fifth contrasts were 1.98, 1.87, 1.25, 1.03 and 0.85 eigenvalue units respectively. The fact that the observed raw variance explained by the measures was ≥ 50% and all contrasts were < 2.0 eigenvalue units further suggested that there was no evidence of multidimensionality.

**Table 2:** Item fit statistics for the Catquest-9SF.

Item	Infit		Outfit		Item calibration^a^ (SE)	DIF Preop to Postop^b^
	MNSQ	ZSTD	MNSQ	ZSTD		
**A—Difficulties in any way in daily life**	0.66	-0.77	0.66	-0.27	-0.74 (0.14)	**-1.01** *
**B—Satisfaction with vision**	0.93	-1.70	0.93	4.40	-2.70 (0.13)	-0.71
**C1—Reading text in newspaper**	1.36	6.37	1.37	5.62	-0.97 (0.14)	**2.06** *
**C2—Recognizing faces**	1.05	-0.50	1.04	-1.11	1.00 (0.16)	**-1.13** *
**C3—Seeing prices**	0.94	-0.65	0.93	-0.81	0.45 (0.15)	0.64
**C4—Seeing to walk on uneven ground**	0.88	-0.77	0.88	-1.30	1.23 (0.16)	-1.08
**C5—Seeing to do needlework, handiwork, carpentry, etc.**	1.09	-1.23	1.08	1.27	0.21 (0.15)	0.64
**C6—Reading text on TV**	0.86	-1.07	0.84	-0.44	0.52 (0.15)	-0.03
**C7—Seeing to carry out a preferred hobby**	0.70	-3.02	0.70	-2.78	1.01 (0.16)	-0.24

DIF: Differential Item Functioning, MNSQ: Mean Square, SE: Standard Error, ZSTD: Standardized Fit Statistic

^a^Measured in logit; an item with positive logit value is an easier item (requires a lower visual ability than the mean of the items); while an item with negative logit value is a more difficult item (requires a higher visual ability than the mean of the items)

^b^Welch’s test, diff (DIF contrast): difference of DIF size between the two subgroups in logits

*p-value < 0.05

### Separation

The Rasch analysis showed good precision and separation ability, with a PSI of 3.28 (> 3.00), and a PR of 0.92 (> 0.9).

### Threshold order

**Fig 1:**
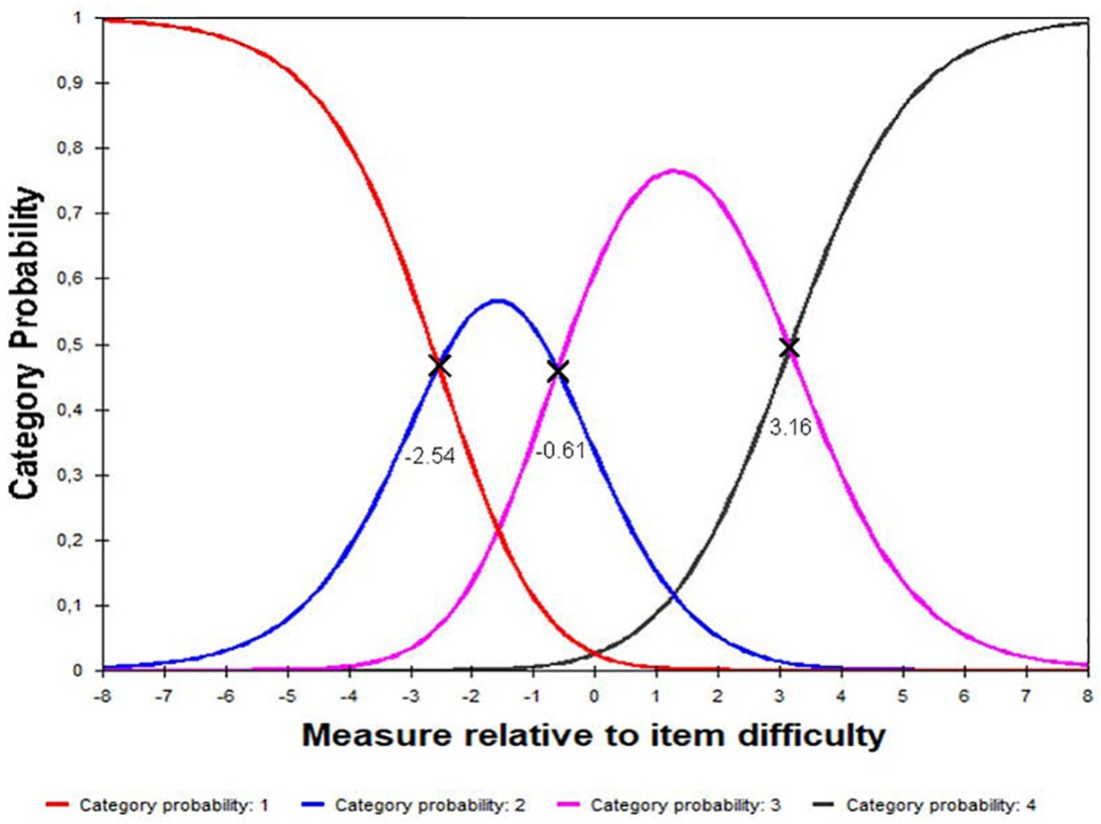
Rating scale category probability curve for the 9-items of Greek-language version.

The category probability curves for the 9-items in Catquest-9SF (Fig 1) illustrate that the category thresholds were ordered for both kinds of response options (A & C1–7 and B), indicating that the original rating scale functioned well. Four response categories were found for all items, suggesting three thresholds for each item [observed average measures for Thurstone thresholds (Standard error): -2.54 (0.11), -0.61 (0.15), 3.16 (0.25)].

### Person-item map

**Fig 2:**
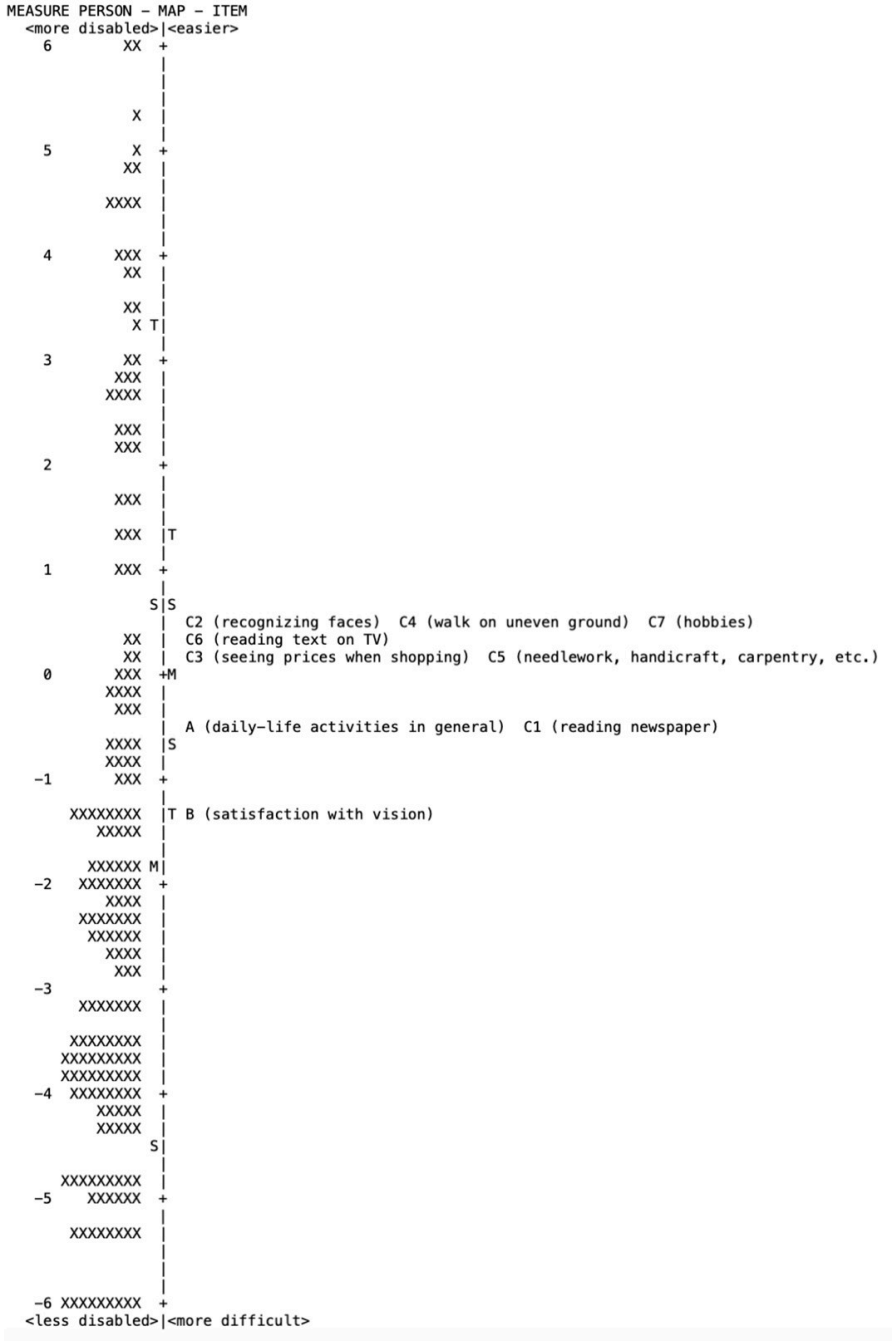
Person-item map of Greek-language version for Catquest-9SF. Each ‘x’ represents 1 respondent. (M = mean; S = 1 standard deviation; T = 2 standard deviations).

The person-item map presented in Fig 2 illustrates the relationship between item difficulty and person ability, and visualizes whether the item difficulties targeted the person abilities in the study sample. The participants are located on the left of the dashed line, with less disabled participants located at the bottom of the diagram. Items are located on the right of the dashed line, with more difficult items being located at the bottom of the diagram. Person ability demonstrated a 13.49-logit spread (-6.78 to 6.71 logits; mean = -1.83 ± 3.02), while item difficulty demonstrated a 1.88-logit spread (-1.37 to 0.51 logits; mean = 0.00 ± 0.69). Thus, the difference between the item and the person means taking into account the stacked data was -1.83, indicating poor targeting. Analyzing preoperative and postoperative data separately, person ability demonstrated a 11.77-logit spread (mean = -0.29 ± 3.10) preoperatively, and a 7.71-logit spread (mean = -4.42 ± 1.83) postoperatively, while item difficulty demonstrated a 2.69-logit spread (mean = 0.00 ± 0.88) preoperatively, and a 3.41-logit spread (mean = 0.00 ± 1.22) postoperatively. The difference between the item and the person means was -0.29 logits preoperatively (< 1 logit), indicating good targeting, and -4.42 postoperatively, indicating mistargeting (absolute value > 1 logit). The postoperative mistargeting between item difficulty and patient ability suggested that the examined tasks of the questionnaire were relatively easy to perform following the cataract surgery, in other words, the respondents were more able than the difficulty of the items.

The easiest item was C7 (hobbies), meaning that only respondents with very low visual function are unable to carry out their hobbies, while the most difficult item was B (satisfaction with vision), namely visual function does not have to be very low for respondents to state that they are dissatisfied with their vision [29].

Finally, the person-item map and person locations of individual questions of Catquest-9SF showed some clustering. In specific, there was one cluster of three items: C2 (recognizing faces), C4 (walk on uneven ground), C7 (seeing to carry out a preferred hobby), and two more clusters each one of them contained two items C3 (seeing prices), C5 (seeing to do needlework, handiwork, carpentry, etc.), and A (daily-life activities in general), C1 (reading text in newspaper) (Fig 2).

### Differential item functioning

DIF was absent or minimal in all items for ocular comorbidity, cataract surgery in the other eye, and binocular BSCDVA with values ranging between -0.34 and 0.48 (DIF contrast < 1.0 logits in all cases, p > 0.05), as well as for age and ranging from -0.83 to 0.43, and from -0.83 to 0.89, respectively [except for C2 for age (DIF contrast = 1.09, p = 0.052), and for C2 (DIF contrast = 1.41, p = 0.005), C3 (DIF contrast = 1.27, p = 0.008) and C5 (DIF contrast = 1.72, p = 0.0006) for gender]. Moreover, DIF was also evaluated between preoperative and postoperative data. Significant DIF was found in items A, C1 and C2 (DIF contrast > 1.00, p < 0.05), while DIF was absent or minimal in the rest items, as presented in the last column of Table 2. Difficulties in any way in daily life (1.01) and recognizing faces (1.13) was rated easier before surgery, while reading text in newspaper (2.06) was rated easier after surgery. The fact that the Greek Catquest-9SF was almost free of DIF indicates that the items functioned similarly across different subgroups of patients.

### Correlations

Performing a multiple stepwise regression analysis, only the preoperative Catquest-9SF logit score was found to have a strong positive correlation with the algebraic difference between preoperative and postoperative Catquest-9SF logit score (logit_preop_—logit_postop_) (coefficient = 0.798, p < 0.0001). This proved that the higher the preoperative Catquest-9SF logit score, indicating a high difficulty of patients in performing the tasks, the greater the patients’ subjective improvement postoperatively. In addition, a negative correlation was found between the algebraic difference in Catquest-9SF score (logit_preop_—logit_postop_) and the postoperative SE (coefficient = -0.825, p = 0.011). In other words, the lower the difficulty, the higher the subjective improvement of patients. The other variables investigated did not contribute to the model and, thus, were excluded.

**Fig 3:**
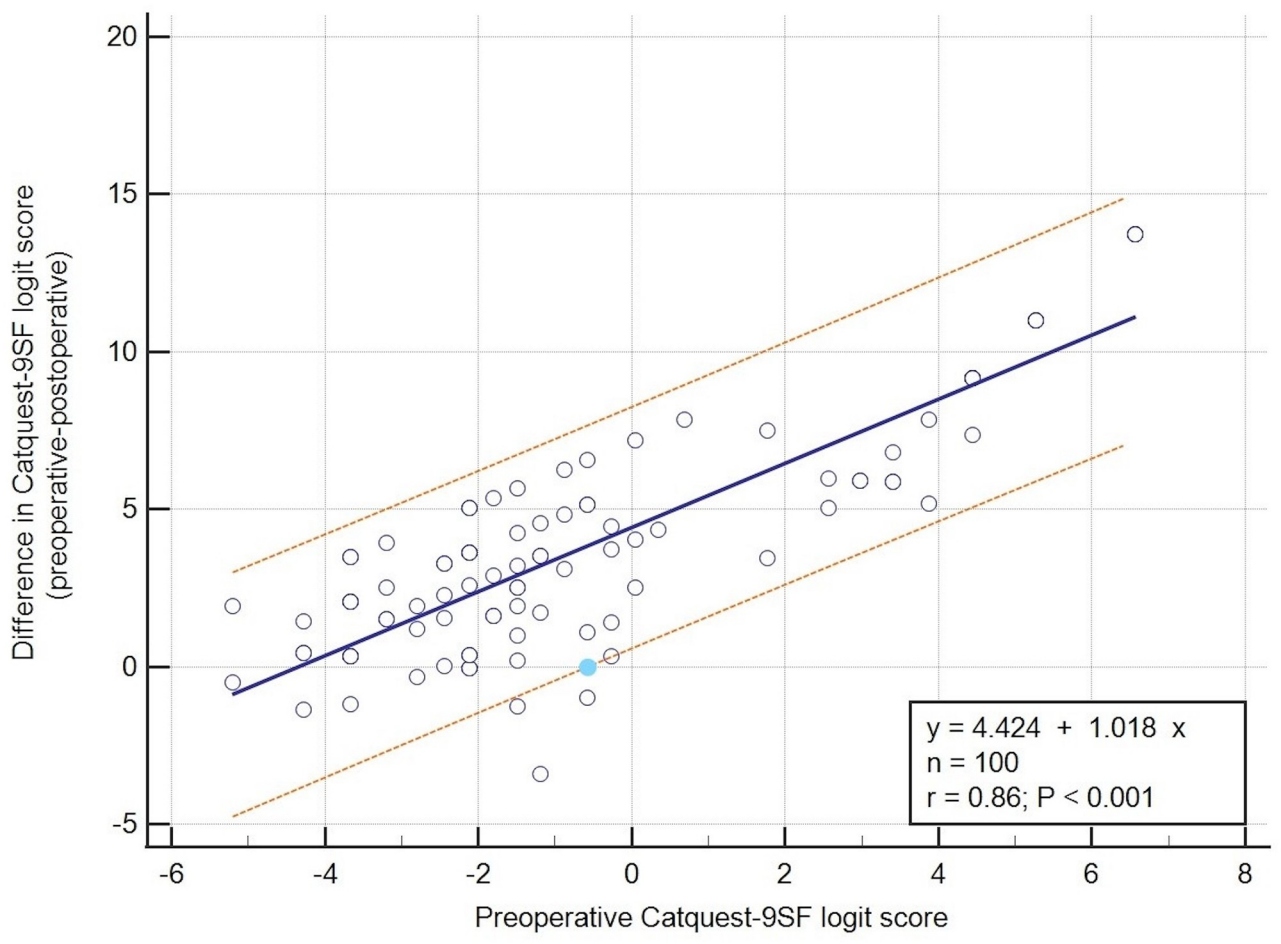
Correlation analysis with 95% prediction interval of the preoperative Catquest-9SF logit score with the difference between the preoperative and the postoperative Catquest-9SF logit score. Line is mean, dotted lines are 95% prediction interval. The blue dot corresponds to the value -0.55.

According to this model, a prediction interval of 95% was performed suggesting that patients who had a preoperative Catquest-9SF logit score of -0.55 or higher had a 95% probability of having a subjective improvement (Fig 3). More specifically, the majority of cases improved and only 9 cases presented a poorer Catquest-9SF score after cataract surgery in comparison with prior to cataract surgery.

Additionally, a significant correlation was found between monocular (in the operated eye) and binocular BSCDVA (preoperative: r = 0.303, p = 0.0021, postoperative: r = 0.886, p < 0.0001). The correlation of monocular (operated and fellow eye) and binocular BSCDVA with logit score preoperatively and postoperatively was also assessed. Only the postoperative BSCDVA of fellow eye was found to be correlated significantly with the postoperative logit score (r = 0.226, p = 0.0238). All correlations examined are presented in Table 3.

**Table 3:** Correlations of BSCDVA with person measures (logit score).

Parameters	r	p value
Preoperative monocular BSCDVA (to be operated eye)—preoperative person measures (logit score)	-0.0816	0.4194
Preoperative binocular BSCDVA—preoperative person measures (logit score)	0.0594	0.5573
Preoperative monocular BSCDVA (fellow eye)—preoperative person measures (logit score)	0.0645	0.5236
Postoperative monocular BSCDVA (operated eye)—postoperative person measures (logit score)	0.119	0.2392
Postoperative binocular BSCDVA—postoperative person measures (logit score)	0.148	0.1422
Postoperative monocular BSCDVA (fellow eye)—postoperative person measures (logit score)	0.226	**0.0238** *

BSCDVA: Best Spectacle-Corrected Distance Visual Acuity

* p value < 0.05

### Test-retest reliability

The ICCs for person ability (person measure in logits) was 0.991 and for item difficulty (item measure in logits) was 0.923, which suggest excellent test–retest reliability of the Catquest-9SF questionnaire.

## Discussion

The Catquest-9SF is one of the most common questionnaires used in daily clinical practice for measuring visual disability, namely vision-related activity limitations [53], on an interval scale. It demonstrates superior psychometric properties and high responsiveness to cataract surgery [40, 55], thus, it has been found that it is appropriate for patients with cataract or following cataract surgery [22, 56].

In our study, the Catquest-9SF was successfully translated into the Greek language and, subsequently, the translated version was psychometrically assessed using Rasch analysis. Catquest-9SF scores were assessed before and following the cataract surgery and a significant improvement in person measures (4.06 logits) was found, similarly to 8 former Catquest validation studies that analyzed both pre- and postoperative data [19, 21–26, 30, 57, 58], showing that the questionnaire is responsive to cataract surgery. Moreover, in our study, the effect size was found to be 1.3, similarly with the effect size of 5 other studies ranging from 1.27 to 2.6 [19, 24, 26, 30, 57, 58]. The fact that the effect size is higher than 0.8 also confirms the responsiveness of the questionnaire. The Greek version of Catquest-9SF presented an excellent repeatability for person ability and item difficulty with high ICC values.

The Greek version of Catquest-9SF showed good precision and separation ability. All nine items of the Catquest-9SF have four response options that signify three ordered thresholds. The ordered thresholds mean that patients who claimed that they had more visual disability for a certain item indeed had more visual disability for that item compared to people who responded that they had less disability. All researchers that previously validated the Catquest-9SF in other languages found adequate precision with PSI ≥ 2.0 and/or PR ≥ 0.80 [19, 21–28, 57–59]. In addition, all previous Catquest validation studies showed ordered thresholds with category probability curves [19, 21–28, 57–59].

As regards unidimensionality assessment, item fit statistics (infit/outfit MNSQ values) derived from our data ranged acceptably between 0.5 and 1.5, and PCA was higher than 50% with unexplained variance in contrasts being lower than 2, indicating the unidimensionality of the questionnaire. Similarly, the majority of previous studies suggested that there was no evidence of multidimensionality, except for two studies [25, 57] which had some misfitting items that were finally excluded from the Rasch analysis. Specifically, Nielsen et al [25] removed item C2 (recognizing faces) (infit = 1.55, outfit = 1.57) and item C4 (seeing to walk on uneven ground) (outfit = 1.68), while Khadka et al. [57] removed item C5 (seeing to do needlework handiwork, carpentry, etc.) (outfit = 1.74).

In the Greek Catquest-9SF, targeting was found to be poor (-1.83 logits) when both preoperative and postoperative data were analyzed. However, targeting was good preoperatively with a difference between the item difficulty and the person ability of -0.29 logits (< 1 logit). On the other hand, mistargeting was present postoperatively with an item-person difference of -4.42 (absolute value > 1 logit). The postoperative mistargeting, which indicates the better ability of respondents after the cataract surgery, is confirmed by all Catquest validation studies that evaluated patient responses postoperatively, which found significant mistargeting ranging between -1.21 [19] and -2.04 [22], or by validation studies that evaluated both preoperative and postoperative patient responses collectively [21, 25, 58]. Negative values indicate that visual function scores improve after surgery and tasks of daily life become easier for respondents to perform. Preoperatively, the questionnaire scale presented a difference between the item difficulty and the person ability from 0.66 to -1.61 in the majority of Catquest validation studies, while 4 studies [21, 22, 27, 59] reported mistargeting ranging from -1.36 to -1.61.

Our study revealed no ceiling or floor effect preoperatively (person measures near zero), giving more “room” for patients to improve their scores, but a marked ceiling effect postoperatively (>15% of respondents achieving the most negative logit score). This could be explained by the improved visual function of patients following the cataract surgery, which leads to a better Catquest score, meaning that questionnaire items become easier postoperatively. The postoperative ceiling effect is probably the cause of mistargeting that appeared after cataract surgery, since a significant number of patients showed no difficulty in daily tasks and had very low logit scores. Validation studies of the French [30], Italian [22], and Spanish [23] version of Catquest-9SF, which evaluated postoperative data, revealed a strong ceiling effect postoperatively, while no ceiling or floor effect was present in the preoperative data of these studies. In contrast, other studies such as the validation studies of the Australian [20] and Chinese versions [27, 28, 57, 59], including only preoperative data, revealed no ceiling or floor effect. Taking into account both preoperative and postoperative data collectively, our study revealed no ceiling or floor effect.

In our study, some clustering was observed, depicted on the person-item maps. Items C2 (recognizing faces), C4 (walk on uneven ground) and C7 (seeing to carry out a hobby) were included in the same cluster. However, item A (daily-life activities in general) was also included in the same cluster with C1 (reading text in newspaper), and C3 (seeing prices) was included in the same cluster with C5 (seeing to do needlework, handiwork, carpentry, etc.). Clustering may imply redundancy of items. Items included in the same cluster likely measure a similar level of visual function. For instance, many people find that recognizing faces may be as difficult as seeing to carry out a preferred hobby. Similar to our study, items C2 and C7 clustered in several studies [22, 28, 57], while in other studies, item C5 (needlework handiwork, carpentry, etc.) clustered, among others, with item C3 (seeing prices) [24, 26, 59]. In addition, the most difficult item in the Greek version of Catquest-9SF was found to be item B (satisfaction with vision), similarly to several studies [19, 20, 29, 58], while the easiest item was C7 (hobbies), in contrast with other studies which showed that C2 (recognizing faces) was the easiest item [19, 20, 29, 58]. In our study, C2 was the third easiest item, while in the study of Lundstrom et al [58] C7 was the second easiest item.

Our questionnaire was free of any large DIF, as confirmed in previous studies of Swedish [19], Australian [20], Chinese and Malaysian cataract patients [28]. However, some evidence of DIF with a few items was found not only in our study, but also in previous ones [19, 20, 24–26, 57, 59]. Nevertheless, no clear patterns were revealed among these results and no notable DIF was detected.

Moreover, a strong positive correlation was found between the difference in Catquest-9SF logit score (logitpreop—logit_postop_) and the preoperative Catquest-9SF logit score, while a strong negative correlation was observed between the difference in Catquest-9SF logit score (logitpreop—logit_postop_) and the postoperative SE. Additionally, Fig 3 of our study shows that only 9% of cases had a worse postoperative Catquest-9SF score than the corresponding preoperative score. This finding is consistent with the research of Lundstrom and Pesudovs [19], in which the majority of cases improved (data appearing below the 1:1 line) and only 9.8% of cases had a poorer postoperative Catquest-9SF score in comparison with the preoperative score. Multiple stepwise regression analysis was also used by the Danish questionnaire validation study, demonstrating a significant correlation between the difference in the Catquest-9SF score and the preoperative logit score, similarly to the present study [25].

As regards correlations with cataract classification, other studies have correlated the Catquest-9SF logit score preoperatively and postoperatively with cataract stage according to LOCS-III classification and localization (Italian version), proving a non-significant correlation [22]. In the present study, all patients had the same cataract stage, so no such correlation was possible. In contrast to our study, Lundstrom et al. in two Swedish multicenter studies showed a significant correlation between the preoperative Catquest-9SF score and the visual acuity of the operated eye and the fellow eye, and a significant correlation between postoperative Catquest-9SF score and the visual acuity of the operated eye [19, 58], while in the one of the aforementioned studies [19], similar to our study, postoperative Catquest-9SF score was found to be correlated also with the visual acuity of the fellow eye. Finally, after examining various combinations of correlations of BSCDVA with person measures, preoperative and postoperative monocular or binocular BSCDVA did not explain clearly the preoperative and postoperative person measure data. Perhaps this could be attributed to the small sample size of our study. However, contrast sensitivity is another important visual impairment measure that could be evaluated in future studies and reveal more clearly the existing correlations between BSCVDA and person measures.

Prior to the interpretation of our results, certain limitations of our study have to be noted. First, all participants were recruited from only two clinical centers located in different regions of Greece. Therefore, in the future, a multicenter clinical study could be conducted including a larger and more representative sample. Second, DIF and correlations of Catquest logit score improvement were not performed with cataract classification since all participants included in the study had a stage 2- senile cataract according to the LOCS-3 grading scale. Thus, patients with a range of cataract severity could be included in future studies and statistical analysis could be expanded in terms of cataract classification. Finally, since a postoperative ceiling effect was present in our data analysis because of the good postoperative visual function, the discrimination of postoperative participants becomes more demanding. To overcome this issue and explore the true range of person measures in normal vision, ophthalmologically normal subjects with normal vision should be included in future studies. Moreover, a larger bank of questions should be created and the questionnaire should be administered via computerized adaptive testing, which is a form of computer-based test that adapts to the respondent’s ability level [56].

## Conclusions

In conclusion, the Catquest-9SF was successfully translated into Greek and presented good psychometric properties. The analysis indicated that the Greek version of Catquest-9SF was reliable, valid, unidimensional and responsive to changes after cataract surgery. The questionnaire scale was found to be well-targeted preoperatively, but a significant mistargeting was found postoperatively, caused by a postoperative ceiling effect, indicating the improved subjective visual ability Catquest scores after surgery. Therefore, this version is a suitable tool for the assessment of the subjective visual functioning in Greek-speaking patients with cataract and following cataract surgery.

## Supporting information

S1 File The original protocol (in Greek) approved by the institutional review board of Democritus University of Thrace. (PDF)

S2 File The original protocol (in Greek) approved by the institutional review board of General Hospital of Kalamata. (PDF)

S3 File The translated protocol (in English) approved by the institutional review board of Democritus University of Thrace. (PDF)

S4 File The translated protocol (in English) approved by the institutional review board of General Hospital of Kalamata. (PDF)

S5 File The Greek version of Catquest-9SF. (PDF)
