# Supplementary material for: Catquest-9SF questionnaire: Validation in a Greek-speaking population using Rasch analysis
Source: PLoS One. 2022 Dec 7;17(12):e0278683. doi: 10.1371/journal.pone.0278683 (PMC9728912; doi:10.1371/journal.pone.0278683)
Supplement: S1 File — (PDF) [file pone.0278683.s001.pdf]

ΕΝΤΥΠΟ ΥΠΟΒΟΛΗΣ ΕΡΕΥΝΗΤΙΚΗΣ ΠΡΟΤΑΣΗΣ ΓΙΑ ΕΓΚΡΙΣΗ ΑΠΟ ΤΟ ΕΠΙΣΤΗΜΟΝΙΚΟ ΣΥΜΒΟΥΛΙΟ ΤΟΥ ΠΑΝΕΠΙΣΤΗΜΙΑΚΟΥ ΓΕΝΙΚΟΥ ΝΟΣΟΚΟΜΕΙΟΥ ΑΛΕΞΑΝΔΡΟΥΠΟΛΗΣ

Εκτίμηση της όρασης σε ασθενείς που υποβάλλονται σε φακοθρυψία με τη χρήση του ερωτηματολόγιου Catquest

Εισαγωγή

Ο καταρράκτης είναι μια από τις πιο διαδεδομένες αιτίες απώλειας όρασης, καθώς ευθύνεται περίπου για το 33% των περιπτώσεων χαμηλής όρασης παγκοσμίως και το 51% των περιστατικών τύφλωσης [1-2]. Η χειρουργική επέμβαση του καταρράκτη είναι μια επέμβαση που εκτελείται συνήθως με υψηλή αποτελεσματικότητα και χαμηλά ποσοστά επιπλοκών [3-5]. Για την καλύτερη δυνατή αξιολόγηση των αποτελεσμάτων της χειρουργικής εξαίρεσης του καταρράκτη, η επιτυχία της επέμβασης θα πρέπει να μετράται τόσο με αντικειμενικό τρόπο, όπως η προεγχειρητική και μετεγχειρητική οπτική οξύτητα και το υπολειπόμενο διαθλαστικό σφάλμα, όσο και με υποκειμενικούς δείκτες, όπως η ικανοποίηση των ασθενών και η ικανότητά τους να εκτελούν δραστηριότητες της καθημερινής ζωής με τη χρήση ειδικών ερωτηματολογίων [6-7]. Αξίζει να σημειωθεί ότι οι υψηλές προσδοκίες ορισμένων ασθενών μετά από μια απρόσκοπτη επέμβαση καταρράκτη μπορεί να οδηγήσουν σε μέτρια ικανοποίηση του ασθενούς παρά την υψηλή μετεγχειρητική οπτική οξύτητα και το ελάχιστο διαθλαστικό σφάλμα, με αποτέλεσμα μία μέτρια οπτική λειτουργία και σχετιζόμενη με την όραση ποιότητα ζωής. [8-9].

Διατίθεται μια ποικιλία εγκυροποιημένων ερωτηματολογίων που αναπτύχθηκαν για την ποσοτική αξιολόγηση των αποτελεσμάτων της χειρουργικής επέμβασης καταρράκτη και της μετεγχειρητικής οπτικής λειτουργίας [10]. Ένα άλλο κοινό εγκυροποιημένο ερωτηματολόγιο για την αυτοαξιολόγηση της οπτικής λειτουργίας είναι το ερωτηματολόγιο Catquest [11]. Αυτό το ερωτηματολόγιο εγκυροποιήθηκε με την ανάλυση κατά Rasch, η οποία είναι η πρότυπη μέθοδος εγκυροποίησης και χρησιμοποιείται για την αξιολόγηση του οφέλους μετά από χειρουργική επέμβαση καταρράκτη. Το ερωτηματολόγιο Catquest αναπτύχθηκε και χρησιμοποιήθηκε για πρώτη φορά στη Σουηδία από το 1995, και το 2009 εγκυροποιήθηκε μια αναθεωρημένη έκδοση 9 σημείων (σύντομη μορφή), που ονομάζεται ερωτηματολόγιο Catquest-9SF. Αν και το ερωτηματολόγιο Catquest είναι διαθέσιμο σε μεγάλη ποικιλία γλωσσών, δεν έχει μεταφραστεί και εγκυροποιηθεί ακόμη στα ελληνικά.

Σκοπός

Ο πρωταρχικός σκοπός της παρούσας μελέτης είναι η εγκυροποίηση του ερωτηματολόγιου Catquest-9SF σε έναν ελληνόφωνο πληθυσμό, η αξιολόγηση στη συνέχεια των ψυχομετρικών του ιδιοτήτων μέσω ανάλυσης κατά Rasch και η αξιολόγηση της προεγχειρητικής και μετεγχειρητικής οπτικής λειτουργίας ασθενών με καταρράκτη χρησιμοποιώντας το ερωτηματολόγιο Catquest πριν και μετά την επέμβαση εξαίρεσης καταρράκτη.

Μέθοδος

Η παρούσα είναι μια προοπτική μελέτη εγκυροποίησης ερωτηματολογίου. Το πρωτόκολλο της μελέτης τηρεί τις αρχές της Διακήρυξης του Ελσίνκι και όλοι οι συμμετέχοντες θα λάβουν γραπτή ενημερωμένη συγκατάθεση. Η μελέτη θα διεξαχθεί στην Οφθαλμολογική Κλινική του Πανεπιστημιακού Γενικού Νοσοκομείου Αλεξανδρούπολης.

Το Catquest-9SF είναι ένα ερωτηματολόγιο για την αξιολόγηση της ποιότητας όρασης των ασθενών με καταρράκτη. Αποτελείται από 9 ερωτήσεις, δύο εκ των οποίων αφορούν την υποκειμενική αξιολόγηση της όρασης του ασθενούς και οι υπόλοιπες επτά αφορούν την ικανότητα του ασθενούς να εκτελεί διάφορες καθημερινές δραστηριότητες.

Το ερωτηματολόγιο Catquest-9SF μεταφράστηκε στην ελληνική γλώσσα από μια μεταφραστική ομάδα αποτελούμενη από πέντε μέλη: έναν συντονιστή μετάφρασης, δύο επαγγελματίες αγγλόφωνους μεταφραστές, έναν επαγγελματία ελληνόφωνο μεταφραστή και έναν Έλληνα οφθαλμίατρο με άριστη γνώση της αγγλικής γλώσσας.

Οι ασθενείς που θα συμμετέχουν στη μελέτη θα κληθούν να συμπληρώσουν ένα ερωτηματολόγιο στα ελληνικά πριν και μετά την επέμβαση καταρράκτη. Τα κριτήρια ένταξης στη μελέτη είναι τα εξής: ηλικία άνω των 18 ετών, διάγνωση ετερόπλευρου ή αμφοτερόπλευρου καταρράκτη με πυρηνική θόλωση σταδίου 2 σύμφωνα με την κλίμακα ταξινόμησης Lens Opacities Classification System III (LOCS-3) και η απουσία σοβαρής γνωστικής διαταραχής κατά την προεγχειρητική εξέταση. Θα συμπεριληφθούν ασθενείς με οφθαλμικές και συστηματικές συννοσηρότητες, καθώς αυτό είναι ένα τυπικό χαρακτηριστικό ενός πληθυσμού με καταρράκτη. Τα κριτήρια αποκλεισμού περιλαμβάνουν τη δυσκολία στην ελληνική γλώσσα ή την κατανόηση της, νευρολογικές ή ψυχιατρικές παθήσεις και γενικά την αδυναμία κατανόησης των ερωτήσεων του ερωτηματολογίου.

Το ερωτηματολόγιο θα διανεμηθεί σε συμμετέχοντες με καλή γνώση της ελληνικής γλώσσας σε γραπτό και προφορικό λόγο. Οι ασθενείς θα συμπληρώσουν την ελληνική έκδοση του Catquest-9SF παρουσία ενός ανεξάρτητου ερευνητή που δεν θα έχει άμεση συμμετοχή στην κλινική εξέταση του ασθενούς. Όλα τα

ερωτηματολόγια θα συμπληρωθούν και θα επιστραφούν την ίδια ημέρα της προεγχειρητικής αξιολόγησης πριν από την κλινική εξέταση. Η προεγχειρητική και μετεγχειρητική αξιολόγηση θα γίνει από τον ίδιο οφθαλμίατρο χωρίς άμεση συμμετοχή στη μελέτη. Τα δημογραφικά και κλινικά δεδομένα των συμμετεχόντων θα ανακτηθούν από τα ιατρικά τους αρχεία. Οι απαντήσεις από συνοδούς ή μέλη της οικογένειας θα εξαιρεθούν. Η επαναληψιμότητα για όλες τις ερωτήσεις του Catquest-9SF θα αξιολογηθεί σε όλους τους συμμετέχοντες σε δύο διαφορετικές επισκέψεις με μεσοδιάστημα 15 ημερών για να αποτραπεί το φαινόμενο μνήμης. Θα αξιολογηθούν οι ακόλουθες κλινικές παράμετροι: η προεγχειρητική και μετεγχειρητική βέλτιστη διορθωμένη με γυαλιά μακρινή οπτική οξύτητα (BSCDVA) του οφθαλμού που έχει προγραμματιστεί για χειρουργική επέμβαση καταρράκτη, η διόφθαλμη BSCDVA, καθώς και η διάθλαση, το σφαιρικό ισοδύναμό και η ενδοφθάλμια πίεση του οφθαλμού που έχει προγραμματιστεί για χειρουργική επέμβαση καταρράκτη.

Μετά την ολοκλήρωση της συλλογής των δεδομένων, αυτά θα υποβληθούν σε επεξεργασία και θα αναλυθούν μέσω της ανάλυσης κατά Rasch για να καθοριστεί, εάν το ερωτηματολόγιο μπορεί να χρησιμοποιηθεί στην κλινική πράξη σε αυτήν τη μεταφρασμένη έκδοση. Η ανάλυση κατά Rasch είναι ένα ψυχομετρικό μοντέλο που βασίζεται στη Κλασική Θεωρία Ελέγχων-Εξετάσεων (CCT), το οποίο χρησιμοποιείται εκτενώς για την αξιολόγηση και τη βελτίωση των υπάρχοντων ερωτηματολογίων [12-18], καθώς και για την κατασκευή νέων ερωτηματολογίων [19-21]. Συγκεκριμένα, η ανάλυση κατά Rasch συγκρίνει το επίπεδο δυσκολίας που απαιτείται για τους ερωτηθέντες για να εκτελέσουν μια εργασία/δραστηριότητα που συμπεριλαμβάνεται στις ερωτήσεις (βαθμός δυσκολίας της ερώτησης – item difficulty) με το επίπεδο ικανότητας των ερωτηθέντων να εκτελέσουν αυτήν τη δραστηριότητα / ερώτηση (ικανότητα των ερωτηθέντων) και αξιολογούνται και τα δύο στην ίδια γραμμική κλίμακα. Οι διατακτικές ακατέργαστες βαθμολογίες των δεδομένων μετατρέπονται σε γραμμικές, ισοδιαστημικές βαθμολογίες Rasch [19, 22], συγκεκριμένα σε μια μονάδα γνωστή ως «logit», η οποία είναι ο δεκαδικός λογάριθμος του λόγου των πιθανοτήτων. Στη συνέχεια, οι βαθμολογίες rasch αθροίζονται σε μια συνολική βαθμολογία για κάθε ερωτώμενο. Αυτή η συνολική βαθμολογία μπορεί να ερμηνευθεί ως μέτρο της λειτουργικής ικανότητας [23]. Η ανάλυση κατά Rasch, στη μελέτη μας, θα πραγματοποιηθεί για την αξιολόγηση των ψυχομετρικών ιδιοτήτων του ερωτηματολογίου Catquest-9SF και θα αξιολογηθεί μέσω παραμέτρων όπως τα κριτήρια προσαρμογής του Rasch, η ανάλυση κύριων συνιστωσών (Principal component analysis-PCA), το κριτήριο διαφορικής απόκρισης των ερωτήσεων (Differential Item Functioning-DIF) κλπ χρησιμοποιώντας την ανάλυση κατά Rasch τόσο για τα προεγχειρητικά όσο και για τα μετεγχειρητικά δεδομένα.

References

1. Pascolini D, Mariotti SP. Global estimates of visual impairment: 2010. Br J Ophthalmol. 2012;5: 614–618. doi: 10.1136/bjophthalmol-2011-300539.
2. National Eye Institute - NEI (2009). Facts about Cataracts. [(Accessed 10 Nov 2019)] Available from: https://www.nei.nih.gov/health/cataract/cataract_facts.
3. Guber I, Rémont L, Bergin C. Predictability of refraction following immediate sequential bilateral cataract surgery (ISBCS) performed under general anaesthesia. Eye Vis (Lond). 2015;2: 13. doi: 10.1186/s40662-015-0023-5.
4. Lundstrom M, Manning S, Barry P, Stenevi U, Henry Y, Rosen P. The European registry of quality outcomes for cataract and refractive surgery (EUREQUO): a database study of trends in volumes, surgical techniques an doutcomes of refractive surgery. Eye Vis (Lond). 2015;2: 8. doi: 10.1186/s40662-015-0019-1.
5. Hodge C, McAlinden C, Lawless M, Chan C, Sutton G, Martin A. Intraocular lens power calculation following laser refractive surgery. Eye Vis (Lond). 2015;2: 7. doi: 10.1186/s40662-015-0017-3.
6. Lamoureux EL, Hassell JB, Keeffe JE. The determinants of participation in activities of daily living in people with impaired vision. Am J Ophthalmol. 2004;137: 265–270. doi: 10.1016/j.ajo.2003.08.003.
7. Chandrasekaran S, Wang JJ, Rochtchina E, Mitchell P. Change in health-related quality of life after cataract surgery in a population-based sample. Eye (Lond). 2008;22: 479–484. doi: 10.1038/sj.eye.6702854.
8. Panagiotopoulou EK, Ntonti P, Vlachou E, Georgantzoglou K, Labiris G. Patients' Expectations in Lens Extraction Surgery: a Systematic Review. Acta Medica (Hradec Kralove). 2018;61: 115-124. doi: 10.14712/18059694.2018.129.
9. Kirwan C, Nolan JM, Stack J, Moore TC, Beatty S. Determinants of patient satisfaction and function related to vision following cataract surgery in eyes with no visually consequential ocular co-morbidity. Graefes Arch Clin Exp Ophthalmol. 2015;253: 1735–1744. doi: 10.1007/s00417-015-3038-7.
10. Massof RW. The measurement of vision disability. Optom Vis Sci. 2002;79: 516–552. doi: 10.1097/00006324-200208000-00015.
11. Steinberg EP, Tielsch JM, Schein OD, Javitt JC, Sharkey P, Cassard SD et al. The VF-14. An index of functional impairment in patients with cataract. Arch Ophthalmol. 1994; 112: 630–638. doi: 10.1001/archopht.1994.01090170074026.
12. Pesudovs K, Garamendi E, Keeves JP, Elliott DB. The Activities of Daily Vision Scale for cataract surgery outcomes: re-evaluating validity with Rasch analysis. Invest Ophthalmol Vis Sci. 2003;44: 2892-2899. doi: 10.1167/iovs.02-1075.
13. Velozo CA, Lai JS, Mallinson T, Hauselman E. Maintaining instrument quality while reducing items: application of Rasch analysis to a self-report of visual function. J Outcome Meas. 2000-2001;4: 667-680.

14. Mallinson T, Stelmack J, Velozo C. A comparison of the separation ratio and coefficient a in the creation of minimum item sets. Med Care. 2004;42: I17–I24. doi: 10.1097/01.mlr.0000103522.78233.c3.
15. Garamendi E, Pesudovs K, Stevens MJ, Elliott DB. The Refractive Status and Vision Profile: evaluation of psychometric properties and comparison of Rasch and summated Likert-scaling. Vision Res. 2006;46: 1375–1383. doi: 10.1016/j.visres.2005.07.007.
16. 34. Lamoureux EL, Pallant JF, Pesudovs K, Hassell JB, Keeffe JE. The Impact of Vision Impairment Questionnaire: an evaluation of its measurement properties using Rasch analysis. Invest Ophthalmol Vis Sci. 2006;47: 4732–4741. doi: 10.1167/iovs.06-0220.
17. McAlinden C, Skiadaresi E, Moore J, Pesudovs K. Subscale assessment of the NEI-RQL-42 questionnaire with Rasch analysis. Invest Ophthalmol Vis Sci. 2011;52: 5685–5694. doi: 10.1167/iovs.10-67951.
18. Khadka J, Pesudovs K, McAlinden C, Vogel M, Kernt M, Hirneiss C. Reengineering the glaucoma quality of life-15 questionnaire with rasch analysis. Invest Ophthalmol Vis Sci. 2011;52: 6971–6977. doi: 10.1167/iovs.11-7423.
19. Bond TG, Fox CM. Applying the Rasch model: fundamental measurement in the human sciences. 2nd ed. New York: Routledge, Taylor & Francis Group; 2007.
20. Pesudovs K, Garamendi E, Elliott DB. The Quality of Life Impact of Refractive Correction (QIRC) questionnaire: development and validation. Optom Vis Sci. 2004;81: 769–777. Doi: 10.1097/00006324-200410000-00009.
21. Pesudovs K, Garamendi E, Elliott DB. The Contact Lens Impact on Quality of Life (CLIQ) questionnaire: development and validation. Invest Ophthalmol Vis Sci. 2006;47: 2789–2796. doi: 10.1167/iovs.05-0933.
22. Khadka J, McAlinden C, Pesudovs K. Quality assessment of ophthalmic questionnaires: review and recommendations. Optom Vis Sci. 2013;90: 720–744. doi: 10.1097/OPX.0000000000000001.
23. Wright BD, Linacre JM, Gustafsson JE, Martin-Loff P. Reasonable mean-square fit values. Rasch Meas Trans. 1994;8: 370.
