# Supplementary material for: Catquest-9SF questionnaire: Validation in a Greek-speaking population using Rasch analysis
Source: PLoS One. 2022 Dec 7;17(12):e0278683. doi: 10.1371/journal.pone.0278683 (PMC9728912; doi:10.1371/journal.pone.0278683)
Supplement: S4 File — (PDF) [file pone.0278683.s004.pdf]

RESEARCH PROPOSAL SUBMISSION FORM FOR APPROVAL BY THE REVIEW BOARD OF GENERAL HOSPITAL OF KALAMATA

Assessment of vision in patients undergoing cataract surgery using the Catquest questionnaire

Background

Cataract is one of the most prevalent causes of vision loss, being responsible for approximately 33% of visual incapacity worldwide and 51% of blindness [1-2]. Cataract surgery is a commonly performed operation with high efficacy and low complication rates [3-5]. For the best possible evaluation of cataract extraction surgery outcomes, the success of the operation should be measured both with objective, such as preoperative and postoperative visual acuity and residual refractive error, and subjective indices, such as patients' satisfaction and their ability to perform activities of daily living using special questionnaires [6-7]. It is worth noting that high expectations of some patients after an uneventful cataract surgery may lead to a moderate patient satisfaction despite the high postoperative visual acuity and the minimum refractive error, resulting in a moderate self-perceived visual function and vision-related quality of life [8-9].

A variety of validated questionnaires developed for the quantitative assessment of cataract surgery outcomes and postoperative visual function are available [10]. Another common validated questionnaire evaluating self-assessed visual function is the Catquest questionnaire [11]. This questionnaire is validated with Rasch analysis, which is the gold standard of validation methods, and is used to evaluate the benefit after cataract surgery. The Catquest questionnaire has been first developed and used in Sweden since 1995, and, in 2009, a revised 9-item version (short-form), called the Catquest-9SF questionnaire, was validated. Although the Catquest questionnaire is available in a great variety of languages, it has been not translated and validated in Greek, yet.

Aim

The primary objective of the present study is to validate the Catquest-9SF questionnaire in a Greek-speaking population, to subsequently assess its psychometric properties via Rasch analysis and to assess the preoperative and postoperative visual function of cataract patients using the Catquest questionnaire before and following the cataract extraction surgery.

Methods

This is a prospective questionnaire validation study. Study protocol adheres to the tenets of the Helsinki Declaration and written informed consent will be obtained by all participants. The study will be conducted in the Department of Ophthalmology of the General Hospital of Kalamata, Kalamata, Greece.

The Catquest-9SF is a questionnaire for assessing the vision quality of cataract patients. It consists of 9 questions, two of which concern the subjective assessment of the patient's vision and the rest seven questions concern the patient's ability to perform various daily activities.

The Catquest-9SF questionnaire was translated into the Greek language by a translation team consisting of five members: one translation coordinator, two professional native English-speaking translators, one professional native Greek-speaking translator and one native Greek ophthalmologist fluent in English.

Patients participating in the study will be asked to complete a questionnaire in Greek before and after cataract surgery. Eligibility criteria include age over 18 years, diagnosis of unilateral or bilateral senile cataract with stage 2 nuclear opalescence according to the Lens Opacities Classification System III (LOCS-3) grading scale, and no severe cognitive impairment during their preoperative examination. Patients with ocular and systemic comorbidities will be included, since this is a typical characteristic of a cataract population. Exclusion criteria include difficulty with the Greek language or comprehension, neurological, psychiatric or mental diseases, and generally inability to understand the Catquest questions.

The questionnaire will be self-administered to Greek-speaking and writing participants. Patients will respond to the Greek version of Catquest-9SF in the presence of an independent researcher who will have no direct involvement in the provision of care. All questionnaires will be completed and return on the same day of the preoperative assessment prior to the clinical examination. The pre- and postoperative assessment will be performed by the same ophthalmologist with no direct involvement in the study. Demographic and clinical data regarding the participants will be retrieved from their medical records. Proxy responses (i.e. from family members) will be excluded. Test-retest reliability for all Catquest-9SF items will be assessed in all participants in two different visits with an average 15-day time window to prevent memory effect. The following clinical parameters will be evaluated: preoperative and postoperative best spectacle-corrected distance visual acuity (BSCDVA) of the eye scheduled for cataract surgery, binocular BSCDVA, as well as refraction, spherical equivalent and intraocular pressure of the eye scheduled for cataract surgery.

Upon completion of the data collection, data will be processed and analyzed through Rasch analysis to determine whether the questionnaire can be used in clinical practice in this translated version. Rasch analysis is a psychometric model based on modern test theory, which is extensively used for the assessment and improvement of existing questionnaires [12-18], as well as for the construction of new questionnaires [19-21]. In specific, Rasch analysis compares the level of difficulty that is required for respondents to perform a task as listed in the items (items' difficulty) with the respondents' level of ability to perform that task (respondents' ability), and both are evaluated along the same linear scale. The ordinal raw scores of the data are transformed into linear, interval Rasch scores [19, 22], namely into a unit known as "logit", which is the natural logarithm of the odds ratio. Then, rasch scores are summed to a total score for each respondent. This total score can be interpreted as a measure of functional ability [23]. Rasch analysis, in our study, will be performed for the assessment of the Catquest-9SF psychometric properties of the questionnaire and will be evaluated through parameters including response category ordering, item fit statistics, principal components analysis, precision, differential item functioning and targeting using Rasch analysisboth for preoperative and postoperative data.

References

1. Pascolini D, Mariotti SP. Global estimates of visual impairment: 2010. Br J Ophthalmol. 2012;5: 614–618. doi: 10.1136/bjophthalmol-2011-300539.
2. National Eye Institute - NEI (2009). Facts about Cataracts. [(Accessed 10 Nov 2019)] Available from: https://www.nei.nih.gov/health/cataract/cataract_facts.
3. Guber I, Rémont L, Bergin C. Predictability of refraction following immediate sequential bilateral cataract surgery (ISBCS) performed under general anaesthesia. Eye Vis (Lond). 2015;2: 13. doi: 10.1186/s40662-015-0023-5.
4. Lundstrom M, Manning S, Barry P, Stenevi U, Henry Y, Rosen P. The European registry of quality outcomes for cataract and refractive surgery (EUREQUO): a database study of trends in volumes, surgical techniques an doutcomes of refractive surgery. Eye Vis (Lond). 2015;2: 8. doi: 10.1186/s40662-015-0019-1.
5. Hodge C, McAlinden C, Lawless M, Chan C, Sutton G, Martin A. Intraocular lens power calculation following laser refractive surgery. Eye Vis (Lond). 2015;2: 7. doi: 10.1186/s40662-015-0017-3.
6. Lamoureux EL, Hassell JB, Keeffe JE. The determinants of participation in activities of daily living in people with impaired vision. Am J Ophthalmol. 2004;137: 265–270. doi: 10.1016/j.ajo.2003.08.003.
7. Chandrasekaran S, Wang JJ, Rochtchina E, Mitchell P. Change in health-related quality of life after cataract surgery in a population-based sample. Eye (Lond). 2008;22: 479–484. doi: 10.1038/sj.eye.6702854.
8. Panagiotopoulou EK, Ntonti P, Vlachou E, Georgantzoglou K, Labiris G. Patients' Expectations in Lens Extraction Surgery: a Systematic Review. Acta Medica (Hradec Kralove). 2018;61: 115-124. doi: 10.14712/18059694.2018.129.
9. Kirwan C, Nolan JM, Stack J, Moore TC, Beatty S. Determinants of patient satisfaction and function related to vision following cataract surgery in eyes with no visually consequential ocular co-morbidity. Graefes Arch Clin Exp Ophthalmol. 2015;253: 1735–1744. doi: 10.1007/s00417-015-3038-7.
10. Massof RW. The measurement of vision disability. Optom Vis Sci. 2002;79: 516–552. doi: 10.1097/00006324-200208000-00015.
11. Steinberg EP, Tielsch JM, Schein OD, Javitt JC, Sharkey P, Cassard SD et al. The VF-14. An index of functional impairment in patients with cataract. Arch Ophthalmol. 1994; 112: 630–638. doi: 10.1001/archopht.1994.01090170074026.
12. Pesudovs K, Garamendi E, Keeves JP, Elliott DB. The Activities of Daily Vision Scale for cataract surgery outcomes: re-evaluating validity with Rasch analysis. Invest Ophthalmol Vis Sci. 2003;44: 2892-2899. doi: 10.1167/iovs.02-1075.

13. Velozo CA, Lai JS, Mallinson T, Hauselman E. Maintaining instrument quality while reducing items: application of Rasch analysis to a self-report of visual function. J Outcome Meas. 2000-2001;4: 667-680.
14. Mallinson T, Stelmack J, Velozo C. A comparison of the separation ratio and coefficient a in the creation of minimum item sets. Med Care. 2004;42: I17–I24. doi: 10.1097/01.mlr.0000103522.78233.c3.
15. Garamendi E, Pesudovs K, Stevens MJ, Elliott DB. The Refractive Status and Vision Profile: evaluation of psychometric properties and comparison of Rasch and summated Likert-scaling. Vision Res. 2006;46: 1375–1383. doi: 10.1016/j.visres.2005.07.007.
16. 34. Lamoureux EL, Pallant JF, Pesudovs K, Hassell JB, Keeffe JE. The Impact of Vision Impairment Questionnaire: an evaluation of its measurement properties using Rasch analysis. Invest Ophthalmol Vis Sci. 2006;47: 4732–4741. doi: 10.1167/iovs.06-0220.
17. McAlinden C, Skiadaresi E, Moore J, Pesudovs K. Subscale assessment of the NEI-RQL-42 questionnaire with Rasch analysis. Invest Ophthalmol Vis Sci. 2011;52: 5685–5694. doi: 10.1167/iovs.10-67951.
18. Khadka J, Pesudovs K, McAlinden C, Vogel M, Kernt M, Hirneiss C. Reengineering the glaucoma quality of life-15 questionnaire with rasch analysis. Invest Ophthalmol Vis Sci. 2011;52: 6971–6977. doi: 10.1167/iovs.11-7423.
19. Bond TG, Fox CM. Applying the Rasch model: fundamental measurement in the human sciences. 2nd ed. New York: Routledge, Taylor & Francis Group; 2007.
20. Pesudovs K, Garamendi E, Elliott DB. The Quality of Life Impact of Refractive Correction (QIRC) questionnaire: development and validation. Optom Vis Sci. 2004;81: 769–777. Doi: 10.1097/00006324-200410000-00009.
21. Pesudovs K, Garamendi E, Elliott DB. The Contact Lens Impact on Quality of Life (CLIQ) questionnaire: development and validation. Invest Ophthalmol Vis Sci. 2006;47: 2789–2796. doi: 10.1167/iovs.05-0933.
22. Khadka J, McAlinden C, Pesudovs K. Quality assessment of ophthalmic questionnaires: review and recommendations. Optom Vis Sci. 2013;90: 720–744. doi: 10.1097/OPX.0000000000000001.
23. Wright BD, Linacre JM, Gustafsson JE, Martin-Loff P. Reasonable mean-square fit values. Rasch Meas Trans. 1994;8: 370.
