# Supplementary material for: Catquest-9SF questionnaire: Validation in a Greek-speaking population using Rasch analysis
Source: PLoS One. 2022 Dec 7;17(12):e0278683. doi: 10.1371/journal.pone.0278683 (PMC9728912; doi:10.1371/journal.pone.0278683)
Supplement: S5 File — (PDF) [file pone.0278683.s005.pdf]

Α/α: ___________ Κλινική:_________ Ημερομηνία:__/__/_____

Catquest-9SF

Αρχικά ασθενούς:______

Ο σκοπός του ερωτηματολογίου αυτού είναι να προσδιορίσει ποιες δυσκολίες έχετε στην καθημερινή σας ζωή λόγω της μειωμένης όρασης.

Για να μπορέσουμε να αναπτύξουμε την υγειονομική μας περίθαλψη όσο το δυνατόν καλύτερα, θα θέλαμε να απαντήσετε στις ερωτήσεις του ερωτηματολογίου με όσο το δυνατόν μεγαλύτερη ειλικρίνεια μπορείτε.

Το ερωτηματολόγιο περιέχει ερωτήσεις σχετικά με τις δυσκολίες που αντιμετωπίζετε εξαιτίας της μειωμένης όρασής σας κατά τη διάρκεια ορισμένων δραστηριοτήτων της καθημερινότητάς σας. Εάν χρησιμοποιείτε γυαλιά για μακριά ή / και για κοντά, οι ερωτήσεις αφορούν την όρασή σας όταν χρησιμοποιείτε τα καλύτερα γυαλιά σας.

Τα ερωτήματα αυτού του ερωτηματολογίου ισχύουν για την κατάστασή σας τις τελευταίες 4 εβδομάδες.

Η συμπλήρωση του ερωτηματολογίου θα επαναληφθεί σε διάστημα περίπου 1 με 3 μήνες μετά το χειρουργείο σας.

Καθώς απαντάτε τις ερωτήσεις στην επόμενη σελίδα, πρέπει να προσπαθείτε να σκέφτεστε μόνο τις δυσκολίες που μπορεί να σας προκαλέσει η όρασή σας. Εκτιμούμε ότι μπορεί να είναι δύσκολο να αποφασίσετε πόσο σημαντική είναι η όραση για εσάς αν έχετε επίσης άλλα προβλήματα όπως για παράδειγμα πόνοι στις αρθρώσεις ή ζάλη. Θα θέλαμε επίσης να προσπαθήσετε να απαντήσετε πόσο σημαντική πιστεύετε ότι είναι η όρασή σας στην ικανότητά σας να εκτελέσετε τις παρακάτω δραστηριότητες.

Όταν σας ζητηθεί να δηλώσετε τις δυσκολίες σας, έχουμε δώσει τρεις επιλογές απαντήσεων. Αυτές είναι οι εξής: **πολύ μεγάλη δυσκολία, μεγάλη δυσκολία και μερική δυσκολία**. Διαφορετικά άτομα μπορούν να επιλέξουν διαφορετικές απαντήσεις. Προσπαθήστε να δείτε τις τρεις επιλογές απαντήσεων ως τρία ισομεγέθη τμήματα μιας κλίμακας που κυμαίνεται από τη μεγαλύτερη έως τη μικρότερη δυσκολία που προκαλείται από την όρασή σας κατά την εκτέλεση διαφόρων δραστηριοτήτων.

Μεγαλύτερη ________________/_______________/_____________ μικρότερη

πολύ μεγάλη δυσκολία μεγάλη δυσκολία μερική δυσκολία

Α. Πιστεύετε ότι η όρασή σας όπως είναι τώρα, σας προκαλεί κάποιας μορφής δυσκολία στην καθημερινότητά σας;

Ναι, πολύ μεγάλη δυσκολία	Ναι, μεγάλη δυσκολία	Ναι, μερική δυσκολία	Όχι, καμία δυσκολία	Δεν μπορώ να αποφασίσω
☐	☐	☐	☐	☐

Β. Είστε ευχαριστημένος ή δυσαρεστημένος με την όρασή σας όπως είναι τώρα;

Πολύ δυσαρεστημένος	Αρκετά δυσαρεστημένος	Αρκετά ευχαριστημένος	Πολύ ευχαριστημένος	Δεν μπορώ να αποφασίσω
☐	☐	☐	☐	☐

Γ. Έχετε δυσκολία με τις παρακάτω δραστηριότητες εξαιτίας της όρασής σας;

Αν ναι, σε τι βαθμό; Σε κάθε σειρά βάλτε μόνο ένα τικ στο κουτί το οποίο πιστεύετε ότι αντιστοιχεί καλύτερα στην κατάστασή σας.

	Ναι, πολύ μεγάλη δυσκολία	Ναι, μεγάλη δυσκολία	Ναι, μερική δυσκολία	Όχι, καμία δυσκολία	Δεν μπορώ να αποφασίσω
Να διαβάζετε κείμενο σε εφημερίδες	☐	☐	☐	☐	☐
Να αναγνωρίζετε τα πρόσωπα των ατόμων που συναντάτε	☐	☐	☐	☐	☐
Να διαβάζετε τις τιμές των προϊόντων όταν πηγαίνετε για ψώνια	☐	☐	☐	☐	☐
Να βλέπετε καθώς περπατάτε σε ανώμαλες επιφάνειες, πχ. λιθόστρωτα	☐	☐	☐	☐	☐
Να βλέπετε να κάνετε κεντήματα, χειροτεχνίες, ξύλινες κατασκευές κλπ.	☐	☐	☐	☐	☐
Να διαβάζετε τους υπότιτλους στην τηλεόραση	☐	☐	☐	☐	☐
Να βλέπετε αρκετά ώστε να συμμετέχετε σε μια δραστηριότητα / χόμπι που σας ενδιαφέρει	☐	☐	☐	☐	☐

Ευχαριστούμε πολύ για τη συμμετοχή σας.
